# Platelet–Neutrophil Interaction and Thromboinflammation in Diabetes: Considerations for Novel Therapeutic Approaches

**DOI:** 10.1161/JAHA.122.027071

**Published:** 2022-10-17

**Authors:** Julia S. Gauer, Ramzi A. Ajjan, Robert A. S. Ariëns

**Affiliations:** ^1^ Discovery and Translational Science Department Institute of Cardiovascular and Metabolic Medicine, University of Leeds Leeds United Kingdom

**Keywords:** diabetes, neutrophils, platelet, thromboinflammation, Inflammation, Platelets

## Abstract

Thromboinflammation has become a topic of key interest in cardiovascular disease and the prevention of diabetes complications because of the interplay between thrombosis and inflammation in diabetes. Specifically, the significant risk of vascular thrombotic disease in diabetes highlights the need for new and better therapeutic targets to help manage and prevent vascular thrombo‐occlusive disease in this condition. Similarly, the prominent role of inflammation in diabetes has sparked interest in anti‐inflammatory agents to better prevent and control vascular disease. Investigations on the effects of anticoagulation and antiplatelet interventions in patients with diabetes and cardiovascular disease show a potential role for these agents in decreasing morbidity and mortality. Neutrophils and platelets are key players in inflammation and wound‐healing response, respectively. The interaction between neutrophils and platelets is thought to be an important driver of thromboinflammation. Therefore, this review describes the mechanisms involved in platelet–neutrophil interactions that contribute to the development or exacerbation of thromboinflammation in the context of diabetes and its associated comorbidities. The effects observed by the antithrombotic/antidiabetic treatments and physical activity/dietary interventions on attenuating thromboinflammation are discussed. These data suggest that mechanisms involved in platelet–neutrophil interaction, platelet activation/aggregation, and the recruitment of neutrophils have a promising potential to become therapeutic targets to decrease thromboinflammation in patients with diabetes.

**Nonstandard Abbreviations and Acronyms**
GD: gestational diabetes
NLR: neutrophil:lymphocyte ratio
T2D: type 2 diabetes
NETs: neutrophil extracellular traps

Thromboinflammation refers to the development of thrombosis that results from an inflammatory state. The interaction between thrombotic and inflammatory responses plays a central role in cardiovascular disease (CVD). However, although patients with diabetes have a higher risk of CVD, the role of thromboinflammation in this population is less well established than in other patient groups, such as those with stroke or coronary artery disease without diabetes.^1^, ^2^ Patients with diabetes have an altered inflammatory state and are at higher risk of developing thrombosis, suggesting that targeting mechanisms associated with both responses, that is, with thromboinflammation, may have important benefits for disease treatment and prevention in these patients. In this review, we explore recent data on the role of the interaction between platelet and neutrophils, key respective players in thrombosis and immune responses, in the development of thromboinflammation in the context of diabetes, and its comorbidities.

## Thromboinflammation in Diabetes

**Figure 1:**
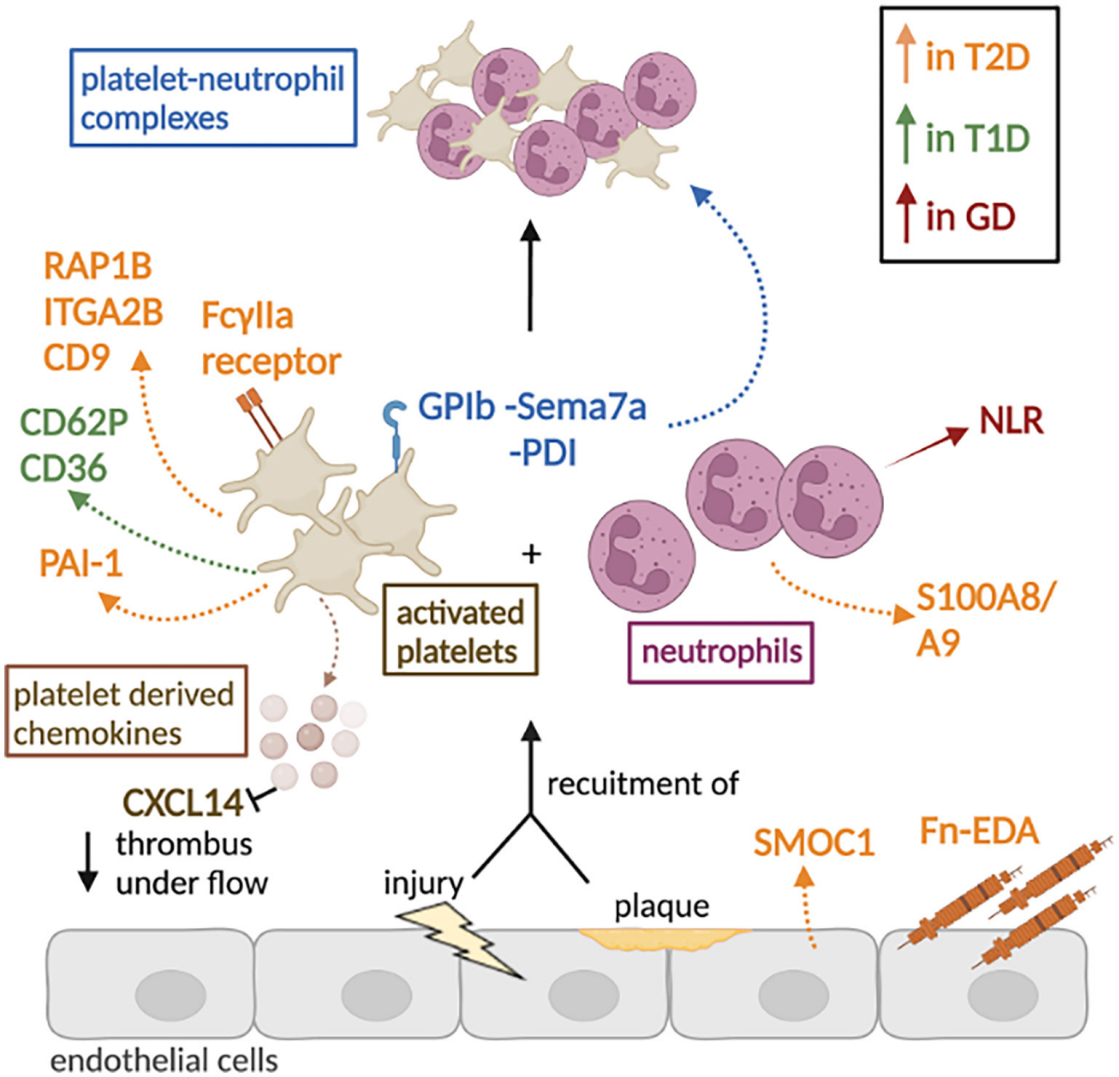
Cellular mechanisms involved in diabetes‐associated thromboinflammation. This figure highlights the mechanisms involved in platelet activation/aggregation and the recruitment of neutrophils that have the potential to become therapeutic targets to decrease thromboinflammation in patients with diabetes. ↑ represents an increase; ↓ represents a decrease. Increased expression of proteins is depicted in orange for type 2 diabetes (T2D), green for type 1 diabetes (T1D), and dark red for gestational diabetes (GD). Upon vessel wall injury or atherosclerotic plaque rupture, platelets are recruited to the site of injury and become activated while neutrophils scout for inflammatory signals and aggregate with amplification of response. Fn‐EDA (fibronectin‐splice variant containing an extra domain A) and extracellular matrix component SMOC1 (secreted modular calcium‐binding protein 1) are increased in T2D. Proteins involved in platelet activation (RAP1B [Ras‐related protein 1b], ITGA2B [integrin subunit alpha 2b], CD9 [CD9 antigen], and Fcγlla [Fcγ receptor type IIa]) are also upregulated in T2D. Similarly, platelet activation markers CD62P (P‐selectin) and CD36 (platelet glycoprotein 4) are upregulated in T1D, suggesting that these proteins could be potential targets to decrease thromboinflammation in T2D and T1D. Other potential targets are platelet‐derived chemokines, for thrombus formation under flow was shown to be decreased following the inhibition of CXCL14 (chemokine C‐X‐C motif ligand 14). Similarly, S100A8/A9 (S100 calcium‐binding proteins A8/A9) calcium‐binding proteins involved in neutrophil chemokine production and aggregation are upregulated in T2D. The neutrophil:lymphocyte ratio (NLR) is reportedly higher in GD and could be used to infer treatment of this condition. Furthermore, increased platelet–neutrophil complexes have been reported by platelet GPIb (glycoprotein 1b) binding to neuronal guidance protein Sema7a (semaphorin 7A) and platelet‐derived PDI (protein disulfide‐isomerase), interactions that could be investigated as potential therapeutic targets. PAI‐1 indicates plasminogen activator inhibitor 1. Image created with BioRender.com.

Platelet hyperactivity is typically observed in diabetes and plays a key role in thromboinflammation.^1^ Increased platelet activity is considered a risk factor for atherosclerosis,^3^ which is accelerated in hyperlipidemic mice with hyper‐reactive platelets.^4^ It has been suggested that platelets of patients with diabetes show increased capability to mediate microvascular thrombosis and inflammation during ischemia/reperfusion injury.^5^ One mechanism at play in diabetes may involve cellular fibronectin, an extracellular matrix glycoprotein that is involved in platelet adhesion to the subendothelium of damaged vessels^6^ (Table 1). Cellular fibronectin is a dimer of Fn‐EDA (fibronectin‐splice variant containing an extra domain A) or Fn‐EDB (fibronectin‐splice variant containing an extra domain B) or varying distributions of both.^7^ TGF‐β (transforming growth factor beta), an inducer of Fn‐EDA splicing and extracellular matrix deposition, is activated in diabetic vessels.^8^ Accordingly, patients with diabetes show higher levels of Fn‐EDA, which promotes thromboinflammation in the context of stroke.^7^, ^9^ *ApoE* ^*−/−*^ mice deficient in Fn‐EDA showed smaller infarct size and reduced inflammatory response following ischemia/reperfusion injury.^7^ Further research is required to determine whether Fn‐EDA could be used as biomarker assessing thromboinflammation in patients with diabetes (Figure 1). Another potential player in the development of thromboinflammation in diabetes is platelet‐derived CXCL14 (chemokine C‐X‐C motif ligand 14), which was shown to have proinflammatory properties through its involvement in isolated human monocyte migration.^10^ Platelets from *apoE* ^*−/−*^ mice deficient in platelet‐specific junction adhesion molecule A, an inhibitor of integrin αIIbβ3 outside‐in signaling, released more CXCL14, suggesting an important role for this chemokine in vascular inflammation and atherosclerosis.^4^ Another study proposed a simplified thromboinflammatory score based on white blood cell count and mean platelet volume:platelet count ratio, for the prognosis of patients with diabetes and ST‐segment–elevation myocardial infarction (MI).^11^ The study concluded that higher scores were associated with higher in‐hospital and 12‐month mortality, suggesting that this scoring system could be used to predict worse immediate and long‐term outcomes.^11^ Furthermore, this study suggests that as well as being useful as an initial prognosis predictor, a measure of thromboinflammation could potentially help establish treatment plans.

**Table 1:** Proteins and Their Observed Effects With Potential to Modulate Diabetes‐Associated Thromboinflammation

	Full or alternative name	Primary role	Effect	References
Fn‐EDA	Fibronectin‐splice variant containing an extra domain A	Involved in platelet adhesion to the subendothelium	↑ in patients with T2D	[9]
			↓ infarct volume in deficient *apoE* ^*−/−*^ mice	[7]
CXCL14	chemokine C‐X‐C motif ligand 14	Platelet‐derived chemokine	↑ plasma levels in the absence of JAM‐A	[4]
			Inhibition ↓ thrombus formation under flow in mice	[51]
PAI‐1	Plasminogen activator inhibitor 1	Inhibitor of tPA	↑ in patients with T2D	[12]
			↑ in hypoglycemia in patients with T1D	[32]
S100A8/A9	S100 calcium‐binding proteins A8/A9	Neutrophil aggregation and chemokine production	↑ in newly diagnosed T2D and hyperglycemia	[26, 27]
			↓ IL‐6 in deficient diabetic mice	[28]
			Blockade ↓ atherosclerosis lesion size in *apoE* ^*−/−*^ mice	[28]
NF‐κB, IL‐6, TNF‐α	Nuclear factor kappa B, interleukin 6, tumor necrosis factor alpha	Transcription factor and markers involved in inflammation	↑ in cultured murine cerebral endothelial cells under hyperglycemia	[25]
			↑ IL‐6 in hypoglycemia in patients with T1D	[32]
			↑ IL‐6 in hyperglycemia following hypoglycemia in patients with T1D	[34]
CD40L	CD40 ligand	Tumor necrosis factor receptor superfamily member, B cell surface antigen	↑ in hypoglycemia in patients with T1D	[33]
FcγIIa	Fcγ receptor type IIa	Involved in platelet activation	↑ in patients with diabetes	[38]
			↑ in inflammatory state	
CD62P, CD36	P‐selectin, platelet glycoprotein 4	Platelet activation markers	↑ P‐selectin in hypoglycemia in	[32]
			patients with T1D	[24]
			↑ lipid levels in patients with T1D	
RAP1B, ITGA2B, CD9	Ras‐related protein Rap‐1b, integrin subunit alpha 2b, CD9 antigen	Involved in platelet activation	↑ in patients newly diagnosed with T2D	[26]
GPIb	glycoprotein 1 b (CD42)	Involved in platelet adhesion	PNC formation via Sema7a binding in samples from patients with MI	[40]
Sema7a	Semaphorin 7A	Neuronal guidance	PNC formation via GPIb binding in samples from patients with MI	[40]
			↑ in patients with MI	
			↓ infarct size in deficient murine models	
CD39	E‐NTPDase	Immune response regulator	↑ thrombi size, leukocyte recruitment to thrombi, and platelet–leukocyte aggregates in deficient mice	[42]
SMOC1	Secreted modular calcium‐binding protein 1	Matricellular component of extracellular matrix	↑ in patients with T2D	[52]
			Hyperactivity to thrombin in mice and humans	[53]
PAD4	Peptidylarginine deiminases 4	Conversion of arginine to citrulline	Inhibition by cl‐amidine ↓ TD1M nonobese diabetic mice	[61]
PDI (platelet derived)	Protein disulfide‐isomerase	Catalyzes formation and breakage of disulfide bonds	↑ PNC formation via GPIbα binding in mice	[65]

↑ represents an increase; ↓ represents a decrease. E‐NTPDase indicates ectonucleoside triphosphate diphosphohydrolase; JAM‐A, junctional adhesion molecule A; MI, myocardial infarction; PNC, platelet–neutrophil complexes; T1D, type 1 diabetes; T2D, type 2 diabetes; and tPA, tissue plasminogen activator.

A further potential biomarker for thromboinfammation in diabetes is PAI‐1 (plasminogen activator inhibitor 1) (Table 1). PAI‐1 reduces the rate of fibrinolysis as it is an inhibitor of tPA (tissue plasminogen activator), which converts plasminogen into plasmin, the latter responsible for the breakdown of fibrin into its degradation products.^12^ PAI‐1 is released from activated platelets and is also expressed by obese adipose tissue.^12^ Increased PAI‐1 levels are found in patients with type 2 diabetes (T2D), which are associated with a hypofibrinolytic state and thromboembolic complications in these patients.^12^ It has been proposed that PAI‐1 could be a potential viable drug target benefiting a variety of diseases.^12^ Increased PAI‐1 levels, alongside evidence that viral RNA fragments and cytokine storm may contribute to insulin resistance,^13^ also shed light on the increased risk of thromboinflammation, morbidity, and mortality in patients with diabetes and COVID‐19.^14^ Another link between thromboinflammation and diabetes was observed in a meta‐analysis showing higher neutrophil:lymphocyte ratio (NLR) in gestational diabetes (GD).^15^ Thrombi retrieved from patients with stroke with high NLR showed increased levels of neutrophil extracellular traps (NETs) marker citrullinated histone 3 compared with samples with low NLR, indicative of a possible association between NLR and NET production in stroke.^16^ NLR was also increased in patients with acute venous thromboembolism,^17^ suggesting that this marker could be used as a tool to inform treatment for GD, venous thromboembolism, and stroke. It should be noted that women with a history of GD are at an increased future cardiovascular risk,^18^ and therefore the documented early thromboinflammatory changes are likely to play a role in the future vascular risk in this population.

## Thromboinflammation and Hyperglycemia

Hyperglycemia was shown to prime thromboinflammatory responses by increasing platelet activation/adhesion and by promoting the release of neutrophil‐ and platelet‐derived microparticles in human and murine samples.^19^, ^20^ For instance, an ischemia/reperfusion cerebral injury study of hyperglycemic mice showed preactivation of platelets and neutrophils, the latter also being more adherent to endothelial cells in hyperglycemic samples from patients with type I diabetes (T1D).^21^ The authors suggested that targeting downstream microvascular thromboinflammation induced by middle cerebral artery occlusion has the potential to decrease the detrimental impact of hyperglycemia in acute ischemic stroke.^21^ Furthermore, hyperglycemia increased infarct size and procoagulant platelet number in mice subjected to transient middle artery occlusion, highlighting the impact of increased glucose levels on stroke risk.^22^ A retrospective study of patients with acute ischemic stroke observed that persistent hyperglycemia was a predictor of poor outcome.^23^

Hyperglycemia also impacted platelet activation in a small‐scale study of patients with T1D, which showed elevated platelet activation markers and platelet–monocyte aggregates compared with nondiabetic controls.^24^ Results indicated that increased platelet activation associated with hyperglycemia in T1D contributes to thromboinflammation and CVD.^24^ A potential mechanism for increased thromboinflammation in diabetes is increased activity of transcription factor NF‐κB (nuclear factor kappa B) and upregulation of genes encoding for several inflammatory markers, including IL‐6 (interleukin 6) and TNF‐α (tumor necrosis factor alpha), as observed in cultured murine cerebral endothelial cells under hyperglycemic conditions^25^ (Table 1). Levels of neutrophil S100A8/A9 (S100 calcium‐binding proteins A8/A9), involved in neutrophil aggregation and chemokine production, were also elevated in patients with T2D in a hyperglycemic state, suggesting that this mechanism may also contribute to the development of thrombinflammation.^26^, ^27^ Furthermore, patients with T2D presented with reticulated thrombocytosis, which occurs in individuals with increased thrombopoietic and megakaryocyte activity.^28^ Thus, targeting the increased levels of cytokines implicated in inflammatory thrombocytosis or platelet production regulatory mechanisms could have great therapeutic value^27^, ^28^ (Figure 1).

## Thromboinflammation and Hypoglycemia

Pharmacological interventions to target the metabolism associated with thromboinflammation in ischemia in the context of hypoglycemia have also been explored. For instance, sodium dichloroacetate was shown to decrease oxidative stress, a trigger for inflammation also involved in the development of venous thrombosis,^29^ and neuronal cell death in hypoglycemia induced by cerebral ischemia in adult male rats.^30^ Nevertheless, although hyperglycemia is associated with an increased thromboinflammatory response,^21^ reducing glucose levels have little effect on vascular events in the short‐medium term, and 1 randomized study showed that intensive glycemic control led to increased mortality in patients with T2D.^31^ One potential reason for this is precipitation of hypoglycemia with aggressive glucose‐lowering therapies, which itself can induce thromboinflammation. Hypoglycemia was shown to induce prothrombotic and proinflammatory mechanisms in healthy individuals and patients with T1D via increased circulating levels of CD62P (P‐selectin), PAI‐1, and IL‐6.^32^ In addition, levels of plasma‐soluble CD40 ligands and platelet–monocyte aggregation were elevated during hypoglycemia in patients with T1D.^33^ Recovery from hypoglycemia has also been implicated in inflammatory mechanisms, with IL‐6 levels elevated in hyperglycemia but not normoglycemia following hypoglycemia in patients with T1D and healthy individuals.^34^ These findings further emphasize the importance of glycemic control, including recovery from hypoglycemia, when considering therapeutic approaches to decrease thromboinflammation in diabetes.

Metabolic disorders, including diabetes, are associated with the overactivation of the sympathetic nervous system (SNS). The SNS has been shown to play a role in the increased oxidative stress commonly associated with features of metabolic syndrome, such as obesity and hypertension.^35^ Moreover, increased activity of coagulation and fibrinolysis pathways has been correlated with SNS overdrive.^36^ Because of the role of the stress axis in inflammatory and thrombotic pathways, overactivity of the SNS and stresses such as hyper‐ and hypoglycemia are important considerations for clinical study design and the interpretation of results.

## Platelet/Neutrophil Alterations as Potential Drivers for Diabetes‐Associated Thromboinflammation

### Increased Platelet Reactivity

Hyperglycemia and hypertriglyceridemia have long been associated with increased platelet reactivity in patients with diabetes.^37^ Expression of FcγIIa (Fcγ receptor type IIa), involved in platelet activation, is elevated in patients with diabetes and in inflammatory states^38^ (Table 1). Platelet activation and systemic inflammation are reportedly correlated in patients with diabetes, as the oxidative stress associated with this condition impairs endothelial cell function, thus decreasing nitric oxide production, leading to platelet activation.^39^ A recent study on patients with T1D showed a higher expression of CD62P (P‐selectin) and CD36 (platelet glycoprotein 4) platelet activation markers correlated with increased lipid levels, suggesting a link between lipid abnormalities and platelet activation.^24^ Gene ontology analysis of plasma microparticles from patients with newly diagnosed T2D revealed the upregulation of the proteins RAP1B (Ras‐related protein 1b), CD9 (CD9 antigen), and ITGA2B (integrin subunit alpha 2b), which appear to play a role in platelet activation.^26^ These findings suggest that blood microparticles may contribute to the hypercoagulable state and thrombotic complications in patients with T2D^26^ (Table 1). Microparticles can be released by a number of cell types, including platelets and leukocytes, and their origin is important for their function.^26^, ^27^ Increased platelet‐derived microparticles in isolated platelets of patients with T2D have been previously reported.^19^

Another player involved in platelet activation implicated in thromboinflammation is GPIb (glycoprotein 1b), which was shown to contribute to platelet–neutrophil complex formation via interaction with the neuronal guidance protein Sema7a (semaphorin 7A) in patients with acute MI^40^ (Table 1). Elevated levels of Sema7a were detected in patients with acute MI and implicated in thrombus formation, as Sema7a‐deficient models developed markedly smaller infarcts.^40^ This mechanism was shown to be GPIb dependent because inhibition of ligand binding to GPIb with a specific Fab fragment abolished the thrombus‐promoting effects of Sema7a at high and low shear rates in mice.^40^ As MI is the leading cause of death in TD2, targeting mechanisms involved in its development could provide critical therapeutic benefits^41^ (Figure 1). One protein with potential benefits in preventing thromboinflammation is CD39 (ectonucleoside triphosphate diphosphohydrolase), as CD39‐deficient mice showed an increased expression of inflammatory mediators, such as CD62P, and larger thrombi under venous stasis^42^ (Table 1). Therefore, this study suggests a potential role for CD39 in subduing thromboinflammation in stasis injury sites, such as venous thromboembolism.^42^

Changes in oxidative phosphorylation have been linked to the platelet activation phase following external stimuli, in human washed platelets.^43^ Recently, increased platelet respiration and altered platelet bioenergetics were identified in murine traumatic brain injury models, suggesting a potential role for platelet bioenergetics in its prognosis and related thromboinflammatory complications.^44^ Furthermore, platelets from healthy individuals with a family history of diabetes showed higher levels of nonmitochondrial respiration compared with individuals without a family history of diabetes, suggesting that mitochondrial bioenergetic profiles could provide an indication of early signs of mitochondrial dysfunction.^45^ Reactive oxygen species, caused by mitochondrial dysfunction, have been linked to the development of inflammation,^46^ and evidence suggests that mitochondrial respiration plays a role in the development of procoagulant platelets.^47^ Altered mitochondrial bioenergetics were also reported in young patients with T1D who showed increased mitochondrial H_2_O_2_ emissions in their skeletal muscles.^48^ Energy production derived from platelet bioenergetics are necessary for platelet aggregation, contraction, and contribution to clot formation, among other mechanical processes.^49^ Interestingly, glucose uptake and glycolysis were increased in a murine streptozotocin T1D model with glucose transporter 3 induction, but platelets of glucose transporter 1 and 3 double‐knockout were unable to use glucose, failing to increase platelet activation in hyperglycemic mice.^50^ In this study, survival after pulmonary embolism was decreased in diabetic mice but increased in double‐knockout mice compared with control.^50^ Because platelet activation is enhanced in hyperglycemia, platelet bioenergetics could be a worthwhile area of investigation in diabetes‐related thromboinflammatory complications.

Chemokines released by platelets represent another area of potential relevance in thromboinflammation, with a recent study showing that platelet‐derived CXCL14 deficiency decreased thrombus formation under flow using murine samples.^51^ A further potential target to modulate platelet hyperresponsiveness in diabetes is SMOC1 (secreted modular calcium‐binding protein 1), which is upregulated in patients with T2D^52^ (Table 1). Platelets of mice deficient in microRNA‐223, a regulator of SMOC1 expression, expressed elevated levels of SMOC1 and presented with hyperactivity to thrombin.^52^, ^53^ Inhibition of SMOC1 in mice and in samples from individuals with T2D with monoclonal antibodies abolished the hyper‐responsiveness of platelets to thrombin, suggesting that this mechanism could be exploited for platelet‐normalizing approaches in patients with diabetes^52^ (Figure 1).

Although most studies concur that patients with diabetes have platelets with altered functionality, 1 study comparing patients with T2D without previous ischemic events matched with individuals without diabetes showed that both groups had comparable CD62P levels, mean platelet volume, and neutrophil–platelet aggregates.^54^ Although little clinical information was provided on the group without diabetes, the disparity between the conclusions of this study and other reports highlights the importance of characterizing underlying mechanisms contributing to altered platelet phenotype in patients with diabetes.

### Altered Neutrophil/NETs Activity

Glucose levels reportedly increase NETs formation (NETosis) in isolated human neutrophils in a concentration‐dependent manner.^55^ Levels of neutrophil elastase and cell free DNA (cfDNA), components of NETs, and homocysteine are elevated in the plasma from patients with diabetes.^56^ Positive correlations between the levels of homocysteine and (1) fasting glucose and (2) NET components elastase and cfDNA suggest that homocysteine may promote NETosis in a diabetic state.^56^ Levels of serum NETs and cfDNA have been shown to be elevated in samples from patients with GD.^57^ The cfDNA levels were also increased posthemodialysis, suggesting that NETosis could contribute to the comorbidities of patients undergoing dialysis.^58^ These are relevant observations as a significant proportion of patients with diabetes develop diabetic kidney disease.^59^

Levels of neutrophils and biomarkers of NETosis, including neutrophil elastase and cfDNA, were elevated in the serum of pediatric patients with recent‐onset T1D, suggesting that altered neutrophil activity leading to increased NETosis at disease onset may be involved in the inflammatory trigger of T1D.^60^ In fact, inhibition of peptidylarginine deiminases 4, primarily expressed in neutrophils and critical in the NETs generation, by cl‐amidine decreased T1D incidence and delayed its onset in nonobese diabetic mice.^61^ Furthermore, proteomic characteristics, determined by gene ontology analysis, of plasma microparticles from patients with T2D showed that levels of S100A8/A9 were upregulated^26^ (Table 1). In addition, the blockade of S100A8/A9 with ABR‐215757 in atherosclerotic‐prone *apoE* ^*−/−*^ diabetic mice decreased atherogenesis, as determined by a reduction in atherosclerotic lesion size and an abundance of plaque macrophages in mice treated with ABR‐215757.^28^ These observations accentuate the role of neutrophils and NETs in the hypercoagulable state associated with diabetes^26^ and therefore their contribution to thromboinflammatory response. In fact, neutrophil tissue factor level was elevated in patients with antineutrophil cytoplasmic antibody‐associated vasculitis, a condition with a reported prevalence of venous thromboembolic events in 1 in 8 patients.^62^, ^63^ In addition, tissue factor was detected in released NETs and circulating microparticles of patients with antibody‐associated vasculitis.^62^ As patients with diabetes are at a 2‐fold higher risk of developing venous thromboembolism, further investigations into the role of neutrophil tissue factor in the context of diabetes could provide useful insights for future therapeutic targets.^64^

### Neutrophil–Platelet Crosstalk

As discussed previously, Sema7a and homocysteine contribute to neutrophil–platelet crosstalk by inducing platelet–neutrophil complex formation and neutrophil/platelet activation, respectively.^40^, ^56^ In addition, thrombi from CD39‐deficient mice were not only larger but also had increased recruitment of leukocytes, and these mice also showed elevated levels of platelet–leukocyte aggregates compared with wild type.^42^ Another protein shown to play a role in platelet–neutrophil interaction is platelet‐derived PDI (protein disulfide‐isomerase) (Table 1).^65^ Through direct binding to platelet GPIbα (glycoprotein Ibα), PDI causes the reduction of 2 allosteric disulfide bonds, augmenting the ligand‐binding activity of GPIbα.^65^ In addition, the number of platelet–neutrophil aggregates (PNAs) were reduced following PDI inhibition under inflammatory (TNF‐α treatment) and thromboinflammatory (sickle cell disease platelets) conditions in murine models.^65^ Interestingly, the number of circulating PDI‐containing microparticles was elevated in samples from patients with T2D, suggesting that mechanisms targeting this protein could have potential benefits in the prevention of thromboinflammatory complications in patients with diabetes.^66^

Increased circulating PNAs were observed in children who are autoantibody positive and children with T1D.^67^ This recent study also found that individuals with lower circulating neutrophil counts, who are at higher risk of developing T1D, had a bigger proportion of neutrophils aggregated to platelets. The authors therefore suggested that PNAs may be useful diagnostic markers for T1D development.^67^ Elevated PNAs and altered platelet bioenergetics have also recently been identified in murine traumatic brain injury models.^44^ As platelet activation and neutrophil–platelet interaction may play a role in thromboinflammatory complications of patients with diabetes, further investigations into the bioenergetics of platelet–neutrophil interaction could have potential therapeutic value. Production of S100A8/A9, mentioned previously, was elevated in the hyperglycemic state in mice.^27^ A follow‐up study with diabetic mice deficient in S100A8/A9 showed lower levels of IL‐6 and reticulated platelets.^28^ Furthermore, the depletion of neutrophils with an anti‐Ly6‐G antibody led to reductions in circulating platelets in diabetic mice, highlighting the involvement of neutrophils in the establishment of thrombopoiesis in diabetes.^28^

An indirect connection between neutrophils and platelets in diabetic complications was presented in a meta‐analysis comparing different parameters in patients with T2D with and without diabetic nephropathy and diabetic retinopathy.^68^ NLR, mean platelet volume, and platelet distribution width were all elevated in patients with diabetic nephropathy/retinopathy.^68^ This study concluded that certain parameters may have potential as diagnostic biomarkers for diabetic nephropathy/retinopathy or other diabetic complications.^68^ Furthermore, a recent meta‐analysis investigating the association of the NLR and platelet:lymphocyte ratio with GD concluded that both parameters were elevated in GD pregnancies when compared with non‐GD pregnancies, albeit only NLR reached statistical significance.^15^ Platelet‐ and neutrophil‐derived microparticles independently mediated glomerular endothelial injury in diabetic nephropathy and were released into the circulation of patients with acute coronary syndrome following coronary intervention.^69^, ^70^ Moreover, higher levels of platelet‐derived microparticles in patients with T2D were associated with leukocyte recruitment via CD62P.^71^ These studies suggest that bioenergetics and platelet/neutrophil microparticles may be useful tools at investigating the development of thromboinflammatory complications in patients with diabetes.

## Targeting Thromboinflammatory Mechanisms—Current Evidence and Potential Benefits

### Current Antithrombotic Drugs

**Figure 2:**
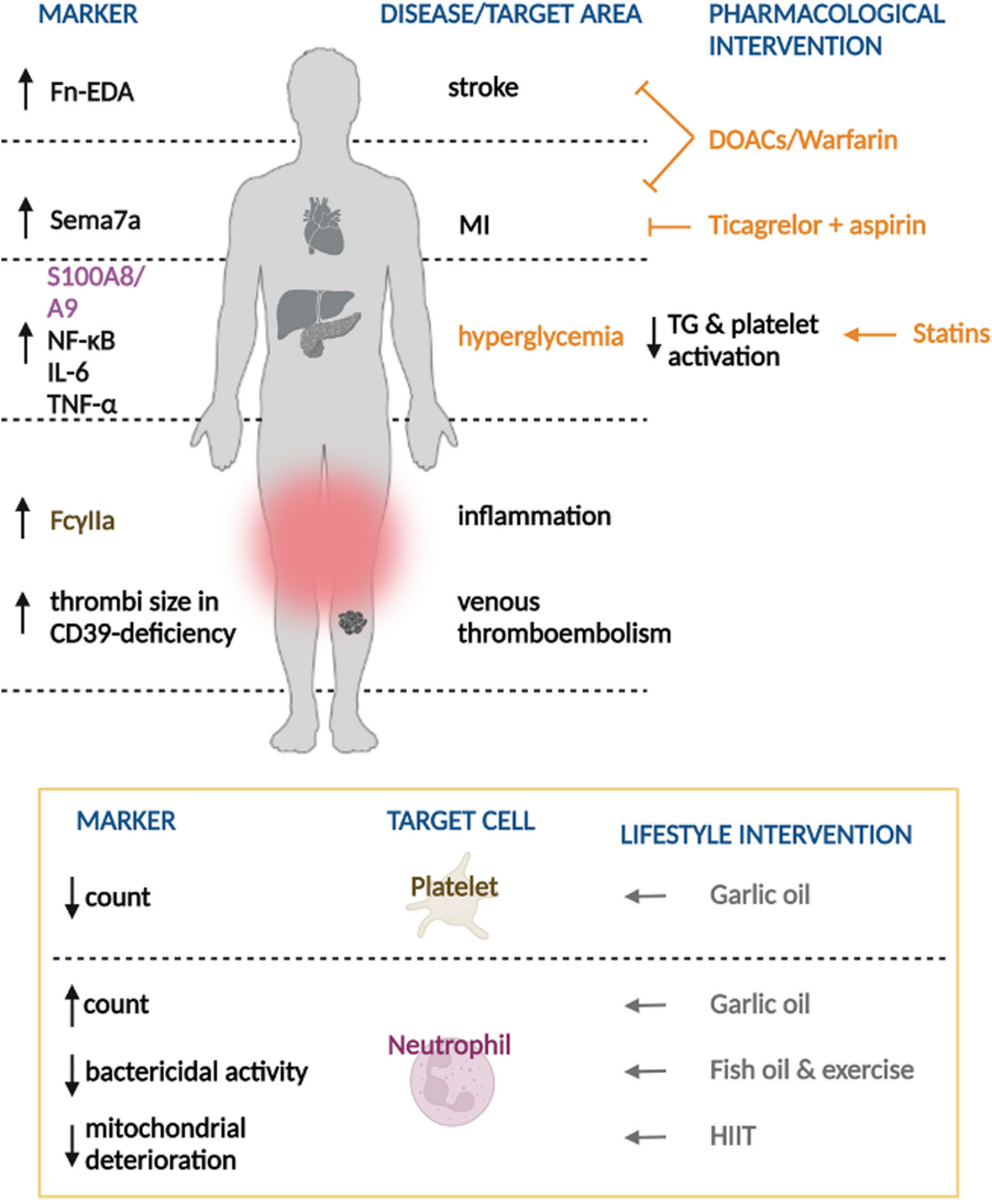
Common health conditions associated with diabetes and the impact of pharmacological, physical activity, and dietary interventions. ↑ Represents an increase; ↓ represents a decrease. The expression of proteins and potential markers in common health conditions associated with diabetes are shown on the left‐hand side. Proteins associated with platelets are shown in brown, and proteins associated with neutrophils are shown in purple. Fn‐EDA (fibronectin‐splice variant containing an extra domain A) is increased in stroke, and neuronal guidance protein Sema7a (semaphorin 7A) is elevated in myocardial infarction (MI). S100A8/A9 (S100 calcium‐binding proteins A8/A9) and inflammation markers NF‐κB (nuclear factor kappa B), IL‐6 (interleukin 6), and TNF‐α (tumor necrosis factor alpha) are increased in hyperglycemia. The expression of platelet receptor Fcγlla (Fcγ receptor type IIa) is increased in inflammation, whereas thrombus size was increased in CD39 (ectonucleoside triphosphate diphosphohydrolase)–deficient mice in the context of venous thromboembolism. Pharmacological interventions that ameliorate each condition in patients with type 2 diabetes (T2D) are shown in orange on the right‐hand side. Direct oral anticoagulants (DOACs)/warfarin decreased the incidence of MI and stroke in patients with T2D with atrial fibrillation. Ticagrelor and aspirin decreased adverse events in patients with T2D and a history of MI. Statins decreased thrombin generation (TG) and platelet activation in patients with T2D. The impact of exercise/dietary interventions on ameliorating platelet/neutrophil activity in the context of diabetes are shown inside the yellow square. Garlic oil decreased platelet count and increased leukocyte count in diabetic rats. Fish oil consumption with regular exercise decreased neutrophil bactericidal activity in sedentary patients who were overweight or obese. A 10‐week, high‐intensity interval training (HIIT) regime decreased neutrophil mitochondrial deterioration in adults with prediabetes. Image created with BioRender.com.

Patients with diabetes are at higher risk of CVD events, and antithrombotic therapies may provide greater clinical benefits to these patients, including the reduction of recurrent events.^72^ For instance, a meta‐analysis comparing the effects of direct oral anticoagulants with vitamin K antagonists, such as warfarin, in patients with diabetes and atrial fibrillation showed that direct oral anticoagulants were more efficacious at preventing stroke, MI, and major bleeding.^73^ Results from the rivaroxaban once daily oral direct factor Xa inhibition compared with vitamin K antagonism for prevention of stroke and embolism trial in atrial fibrillation trial, however, showed similar relative efficacy of warfarin and rivaroxaban for stroke prevention in >5000 patients with T2D with atrial fibrillation.^74^ A recent review provides further insight on antithrombotic therapy in diabetes.^72^ The cardiovascular outcomes for people using anticoagulation strategies trial demonstrated that combined rivaroxaban plus aspirin treatment significantly decreased major vascular events and mortality in stable patients with coronary artery disease^75^ (Table 2). Recently, in addition to their antithrombotic effects, direct oral anticoagulants also showed anti‐inflammatory effects in patients with vein thrombosis by reducing IL‐6 levels.^76^ Further studies are required to determine the safety/efficacy of direct oral anticoagulants, including their anti‐inflammatory effects, in different subgroups of individuals with diabetes given this is a highly heterogeneous population with variable cardiovascular risk (Figure 2).

**Table 2:** Antithrombotic Interventions and Their Observed Effects With Potential to Modulate Thromboinflammation

Intervention	Cohort	Effect	References
DOACs vs warfarin	Patients with diabetes and AF	↓ stroke, major bleeding, and MI	[73, 74]
		Improved efficacy and safety with DOACs	
Rivaroxaban+aspirin	Stable patients with CAD	↓ major vascular events and mortality	[75]
DOACs	Patients with deep vein thrombosis	↓ IL‐6 levels	[76]
Clopidogrel	Patients with recent ischemic stroke, MI, or symptomatic peripheral arterial disease	↓ adverse events in patients with diabetes, particularly in conjunction with insulin therapy	[77]
	Experimental human model	↓ systemic inflammation	[79]
Ticagrelor+aspirin	Patients with diabetes and history of MI	↓ adverse events	[78]
Ticagrelor	Patients with ACS	↓ polyphosphate‐induced NETs	[84]
	Experimental human model	↓ systemic inflammation and prothrombotic clot phenotype	[79]
Inclacumab	Patients with non–ST‐segment–elevation MI	↓ MI	[81]
Canakinumab	Patients with history of MI and elevated C‐reactive protein	↓ cardiovascular event recurrence	[82]
Statins	Patients with T2D	↓ thrombin generation and platelet activation	[85]

↑ represents an increase; ↓ represents a decrease. ACS indicates acute coronary syndrome; AF, atrial fibrillation; CABG, coronary artery bypass draft; CAD, coronary artery disease; CVD, cardiovascular disease; DOACs, direct oral anticoagulants; IL‐6, interleukin 6; MI, myocardial infarction; NETs, neutrophil extracellular traps; and T2D, type 2 diabetes.

Several trials have demonstrated benefits of antiplatelet therapy alone in patients with diabetes and coronary artery disease or acute coronary syndrome.^72^ For instance, the clopidogrel versus aspirin in patients at risk of ischemic event study showed that clopidogrel prevented a higher number of adverse events in patients with diabetes than patients without diabetes.^77^ Recently, the prevention of cardiovascular events in patients with prior heart attack using ticagrelor compared to placebo on a background of aspirin‐thrombolysis in myocardial infarction 54 trial showed that ticagrelor, combined with low‐dose aspirin, reduced the number of adverse events in patients with diabetes and a history of MI compared with a placebo group.^78^ In addition, platelet P2Y_12_ receptor inhibitors, which have well‐established effects on platelet activation inhibition by adenosine diphosphate during thrombosis, were shown to decrease systemic inflammation and its associated prothrombotic effects in an experimental human model of inflammation triggered by endotoxin.^79^ In this study, platelet–monocyte aggregates, proinflammatory cytokines TNFα and IL‐6, and D‐dimer (a fibrin degradation product, indicative of dissolution of existing blood clots) were reduced by clopidogrel and ticagrelor treatment, with ticagrelor also altering the fibrin clot structure to a less prothrombotic phenotype.^79^ Furthermore, clinical trials investigating the benefit of blocking platelet–leukocyte interaction using antibodies against CD62P showed promising results.^80^ For instance, inclacumab, a monoclonal antibody targeting CD62P, was shown to reduce myocardial damage in patients with non–ST‐segment–elevation MI following percutaneous coronary intervention.^81^ Anti‐inflammatory therapy with canakinumab, a monoclonal antibody targeting IL‐1 (interleukin 1), decreased the rate of cardiovascular event recurrence in patients with previous MI and elevated C‐reactive protein levels.^82^ Future investigations are required on therapeutics to influence guidelines on the prevention of thromboinflammation in patients with diabetes. The use of small molecules to interfere with platelet–leukocyte interaction are also being explored as demonstrated by a recent study that identified the minimal sequence of the extracellular fibrinogen‐binding protein from *Staphylococcus aureus* that directly binds to CD62P, thereby inhibiting platelet–leukocyte interaction.^83^ The effects of ticagrelor on inorganic polyphosphates released by thrombin‐activated platelets, which promote NET formation derived by platelet–neutrophil interaction, have also been recently investigated.^84^ Neutrophils from healthy individuals or patients with stent placement receiving ticagrelor following acute coronary syndrome were stimulated to produce NETs.^84^ Ticagrelor was shown to attenuate NETs induced by polyphosphates but did not affect polyphosphate secretion from thrombin‐activated platelets, suggesting that it has the potential to promote an immune‐regulatory effect by attenuating NET formation^84^ (Table 2).

Furthermore, the impact of lipid‐lowering drugs on thromboinflammation risk in patients with diabetes has also been investigated (Figure 2). Procoagulant status, determined by a thrombin‐generation assay, and platelet activation were decreased in patients with T2D following treatment with statins^85^ (Table 2). Statins have been shown to inhibit inflammatory factors including NF‐ĸB,^86^ which is elevated in hyperglycemia,^25^ suggesting a potential to counteract thromboinflammation (Figure 2). This indicates that statins affect both inflammation and thrombosis, thus targeting thromboinflammatory mechanisms that potentially explain reduced vascular events.

As suggested by the studies discussed previously, it is feasible that prophylactic antithrombotic medications could be beneficial at preventing thromboinflammatory events in patients with diabetes. Nevertheless, it is evident that although pharmacological interventions targeting thrombosis and inflammation in isolation have been explored, few have investigated their effects on the thromboinflammatory response of patients with diabetes. As patients with diabetes have an altered inflammatory state and increased risk of thrombosis, assessing the effect of pharmacological therapies on thromboinflammatory response may have benefits regarding drug management. In addition, the development of new therapies targeting the mechanisms involved in platelet–neutrophil crosstalk to prevent and treat thromboinflammation have been suggested to be advantageous over current antithrombotic therapies as normal hemostasis should be impacted less.^87^

### Exercise and Diet

The impact of physical activity on platelet/neutrophil function may provide an important link between habitual exercise and thromboinflammatory risk (Table 3). A study investigating warm‐up exercise and platelet–neutrophil interaction in healthy, sedentary men showed that warm‐up exercise attenuated the increase in platelet–neutrophil aggregation and subsequent oxidative bursts driven by high‐intensity exercise.^88^ In addition, light‐intensity exercise was shown to minimize thromboinflammation by decreasing platelet–neutrophil aggregation.^88^ Several studies have reported an increase in NLR following acute exercise in healthy young^89^ and middle‐aged men^90^ and older individuals with coronary artery disease symptoms.^91^ A study comparing the effect of submaximal exercise on platelet function of moderately active men at different times of day showed that PNAs were higher postevening exercise.^92^ Nevertheless, a recent study on individuals with multiple sclerosis observed a decrease in NLR following high‐intensity interval training.^93^ The same study showed increased platelet:lymphocyte ratio,^93^ however. Increased platelet:lymphocyte ratio was also reported in the aforementioned studies.

**Table 3:** Physical Activity and Dietary Interventions and Their Observed Effects With Potential to Modulate Thromboinflammation

Thromboinflammatory marker	Physical activity/dietary intervention	Effect	References
Platelet–neutrophil aggregates	HIE (40 min, 80% maximal oxygen consumption)	↑ in healthy, sedentary men	[88]
	HIE (40 min, 80% maximal oxygen consumption) following WUE (20 min, 40% maximal oxygen consumption)	↓ in healthy sedentary men	[88]
	LIE (40 min, 40% maximal oxygen consumption)	↓ in healthy sedentary men	[88]
	Submaximal exercise (70% maximal oxygen consumption)	↑ moderately active men	[92]
	Flavanol‐rich cocoa beverage	↓ in healthy volunteers	[95]
Neutrophil:lymphocyte ratio	Acute exercise (>65% maximal oxygen consumption)	↑ healthy young men	[89]
		↑ middle‐aged men	[90]
	Treadmill stress test	↑ older individuals with CAD	[91]
	HIIT (5×1.5 min at 95%–100% maximum heart rate)	↓ in individuals with MS	[93]
Platelet:lymphocyte ratio	Acute exercise (>65% maximal oxygen consumption)	↑ healthy young men	[89]
	Treadmill stress test	↑ older individuals with CAD	[91]
	HIIT (5×1.5 min at 95%–100% maximum heart rate)	↑ in individuals with MS	[93]
	Alfrutamide and caffedymine	↓ in healthy mice	[97]
Neutrophil mitochondrial deterioration	ASE	↑ sedentary young men	[46]
	CME	↓ sedentary young men	[46]
	10‐wk HIIT	↓ older adults with prediabetes	[94]
Platelet count and clotting factors	Garlic oil	↓ diabetic rats	[96]
Leukocyte count	Garlic oil	↑ in diabetic rats	[96]
Neutrophil bactericidal activity	Fish oil supplementation and regular exercise	↓ in nonexercising patients with CVD risk who are overweight or obese	[98]

↑ represents an increase; ↓ represents a decrease. ASE indicates acute severe exercise; CAD, coronary artery disease; CME, chronic moderate exercise; CVD, cardiovascular disease; HIE, high‐intensity exercise; HIIT, high‐intensity interval training; LIE, low‐intensity exercise; MS, multiple sclerosis; and WUE, warm‐up exercise.

Oxidative stress and mitochondrial reactive oxygen species, associated with neutrophil apoptosis, were increased following acute severe exercise in sedentary young men.^46^ Chronic moderate exercise, on the other hand, delayed mitochondrial deterioration and spontaneous neutrophil apoptosis in the same study.^46^ Recently, a study assessing the effect of high‐intensity interval training on diabetes risk in older adults with prediabetes showed that following a 10‐week program, neutrophil mitochondrial function was improved, and basal reactive oxygen species decreased to a level comparable with those observed in young adults.^94^

Platelet/neutrophil function and coagulation can be directly influenced by dietary habits (Table 3). For instance, PNAs in healthy volunteers were decreased following consumption of flavanol‐rich cocoa beverages.^95^ In another study, platelet count and plasma concentrations of clotting factors in diabetic rats were decreased following treatment with garlic oil.^96^ On the other hand, the authors observed increased leukocytes following garlic oil treatment.^96^ The study suggests that garlic oil could provide multicellular modulation of platelet and neutrophils reactivity in addition to functional interactions with plasma coagulation factors and platelet cofactors.^96^ CD62P expression and platelet–leukocyte aggregation were decreased in mice following treatment with alfrutamide and caffedymine, phenolic amides commonly found in cocoa and garlic.^97^ Another study investigating the combined effect of regular exercise in combination with ω‐3 polyunsaturated fatty acid supplementation with fish oil in individuals with CVD risk factors who were overweight or obese showed that neutrophil bactericidal activity was maintained by regular exercise but decreased in nonexercising participants.^98^

The benefits of physical activity and maintaining a healthy diet in CVD and diabetes prevention are well established.^99^ Further investigations into the benefits of warm‐up exercise and exercise intensity, independently or in combination with dietary interventions, at preventing thromboinflammation in patients with or at risk of developing diabetes could aid in the design of targeted physical activity and dietary guidelines for different patient groups (Figure 2).

Thrombosis and inflammation have been associated with increased levels of satiety hormone leptin, commonly observed in patients who are obese who become leptin resistant.^35^ Therefore, diet and exercise may also be important considerations to counteract SNS overdrive and reduce stress‐related thromboinflammatory risk factors for CVD and diabetes as well as improve patient prognosis. Moreover, SNS overdrive, linked with increased oxidative stress and elevated activity of coagulation pathways as previously mentioned, may also be counterbalanced by lifestyle interventions that stimulate the parasympathetic nervous system by eliciting a relaxation response. For instance, a meta‐analysis of studies on individuals with CVD showed that mindfulness‐based interventions were beneficial physically, by decreasing blood pressure and heart rate, as well as psychologically, by alleviating stress, anxiety, and depression.^100^ Likewise, other approaches such as mind–body exercises and proper sleep hygiene may contribute to the improvement of several stress‐related CVD markers and risk factors, suggesting that these lifestyle interventions could have a significant impact on the quality of life and prognosis of patients with diabetes.

## Conclusions

Evidence for a role for thromboinflammation in the pathological mechanisms of diabetes is becoming increasingly paramount. Both hyper‐ and hypoglycemia can trigger cellular mechanisms of thromboinflammation. The hyper‐reactivity of platelets and neutrophils in diabetes contributes to substantial thromboinflammatory stress. Although recent studies suggest improvements in the treatment of thrombosis in diabetes using dual‐antiplatelet therapy and direct oral anticoagulants, further studies are required to investigate the appealing potential of targeting thromboinflammatory mechanisms in diabetes, with initial trials of anti‐CD62P drugs showing promise. Furthermore, both exercise and dietary intervention in patients with diabetes are also appealing to alleviate diabetes‐related thromboinflammatory stress. Mindfulness‐based approaches could also further stimulate physical health and promote psychological well‐being. Future studies in these novel areas are expected to lead to substantial improvements for the treatment of cardiovascular risk in patients with diabetes.

## Sources of Funding

Gauer is supported by a Mautner British Heart Foundation Career Development Fellowship. Research work in the Ajjan group is currently supported by The National Institute for Health and Care Research, Diabetes UK, British Heart Foundation, Biotechnology and Biological Sciences Research Council, and Abbott Diabetes Care. The Ariëns laboratory is supported by grants from the British Heart Foundation (RG/18/11/34036), Wellcome Trust (204 951/B/16/Z), and Biotechnology and Biological Sciences Research Council (BB/W000237/1).

## Disclosures

None.
